# Downregulation of SODD mediates carnosol-induced reduction in cell proliferation in esophageal adenocarcinoma cells

**DOI:** 10.1038/s41598-023-37796-5

**Published:** 2023-06-29

**Authors:** Aihua Li, Weibiao Cao

**Affiliations:** 1 Department of Pathology and Laboratory Medicine, Rhode Island Hospital, The Warren Alpert Medical School of Brown University, 593 Eddy St, APC12, Providence, RI 02903 USA; 2 Department of Gastroenterology, Chongqing University Cancer Hospital, Chongqing, China

**Keywords:** Cancer, Gastroenterology

## Abstract

Esophageal adenocarcinoma carries a poor prognosis associated with a 5-year survival rate of 12.5–20%. Therefore, a new therapeutic modality is needed for this lethal tumor. Carnosol is a phenolic diterpene purified from the herbs such as rosemary and Mountain desert sage and has been shown to have anticancer activities in multiple cancers. In this study we examined the effect of carnosol on cell proliferation in esophageal adenocarcinoma cells. We found that carnosol dose-dependently decreased cell proliferation in FLO-1 esophageal adenocarcinoma cells and significantly increased caspase-3 protein, indicating that carnosol decreases cell proliferation and increases cell apoptosis in FLO-1 cells. Carnosol significantly increased H_2_O_2_ production and N-acetyl cysteine, a reactive oxygen species (ROS) scavenger, significantly inhibited carnosol-induced decrease in cell proliferation, indicating that ROS may mediate carnosol-induced decrease in cell proliferation. Carnosol-induced decrease in cell proliferation was partially reversed by NADPH oxidase inhibitor apocynin, suggesting that NADPH oxidases may be partially involved in carnosol’s effect. In addition, carnosol significantly downregulated SODD protein and mRNA expression and knockdown of SODD significantly inhibited the carnosol-induced reduction in cell proliferation, suggesting that downregulation of SODD may contribute to carnosol-induced reduction in cell proliferation. We conclude that carnosol dose-dependently decreased cell proliferation and significantly increased caspase-3 protein. Carnosol’s effect may be through the overproduction of ROS and the downregulation of SODD. Carnosol might be useful for the treatment of esophageal adenocarcinoma.

## Introduction

Esophageal adenocarcinoma has increased in incidence over the past several decades^1–3^. The major risk factor for esophageal adenocarcinoma is Barrett’s esophagus^4^, which carries nearly a 30–125-fold increased risk for the development of esophageal adenocarcinoma. The cancer incidence in Barrett’s esophagus is about 0.5–1.0% per year. The mechanisms of the progression from Barrett’s esophagus to adenocarcinoma are not fully understood. Many genetic and epigenetic alterations, chromosomal gains, and losses, and hypermethylation of gene promoters may be involved in this progression^5,6^. Esophageal adenocarcinoma has a poor prognosis associated with a median survival of less than 1 year^7,8^ and a 5-year survival rate of 12.5–20%^9,10^. Therefore, a new therapeutic modality is needed for this lethal tumor.

Carnosol is a phenolic diterpene purified from the herbs such as rosemary and Mountain desert sage, and has been shown to have anticancer activities in multiple cancers, including colon^11^, breast^12^, stomach^13^ and prostate^14^. It may inactivate STAT3 through the production of reactive oxygen species (ROS), thus inhibiting tumor migration and growth in breast cancer cells^12^. Carnosol may selectively inhibit the p300 histone acetyl transferase^15^ and triggers a ROS-dependent ER-stress response through activation of the three ER stress sensor pathways in breast cancer cells^16^. ROS dependent inactivation of STAT3 also mediates carnosol-induced apoptosis in human colon cancer HCT116 cells^11^. In gastric cancer, carnosol suppresses gastric cancer growth via inhibiting the RSK-CREB signaling pathway^13^. Whether carnosol has anticancer activity in esophageal adenocarcinoma is not known. The aim of this study is to examine the effect of carnosol on cell proliferation in esophageal adenocarcinoma cells and whether its effect is mediated by the silencer of death domains (SODD).

## Results

### Carnosol reduced cell proliferation and increased cell apoptosis in esophageal adenocarcinoma cells

**Figure 1:**
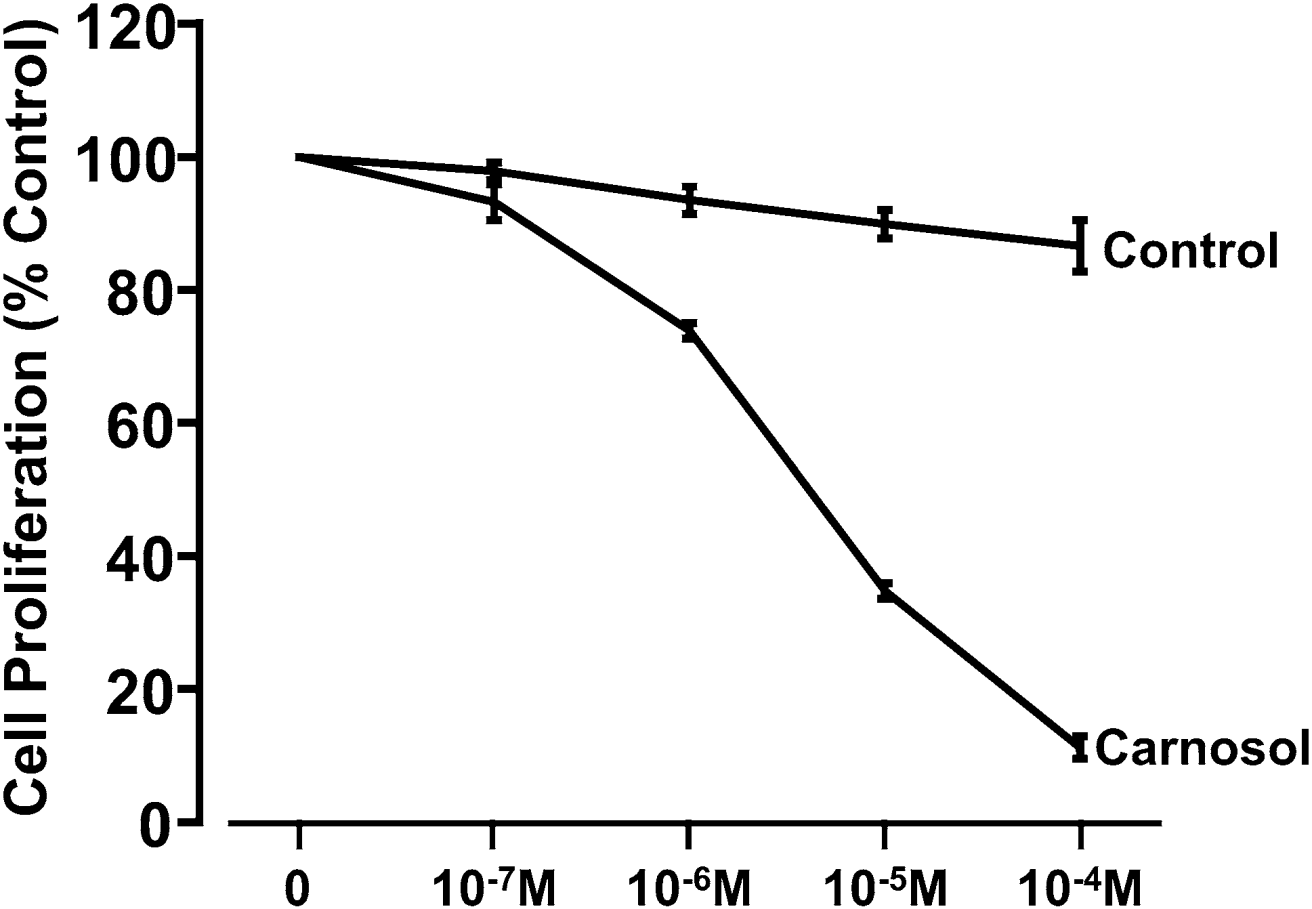
Effect of carnosol on cell proliferation. 24-h treatment with carnosol dose-dependently decreased cell proliferation in esophageal adenocarcinoma cells FLO-1, suggesting that carnosol decreases cell proliferation in FLO-1 cells. N = 3, ANOVA, *P* < 0.001.

**Figure 2:**
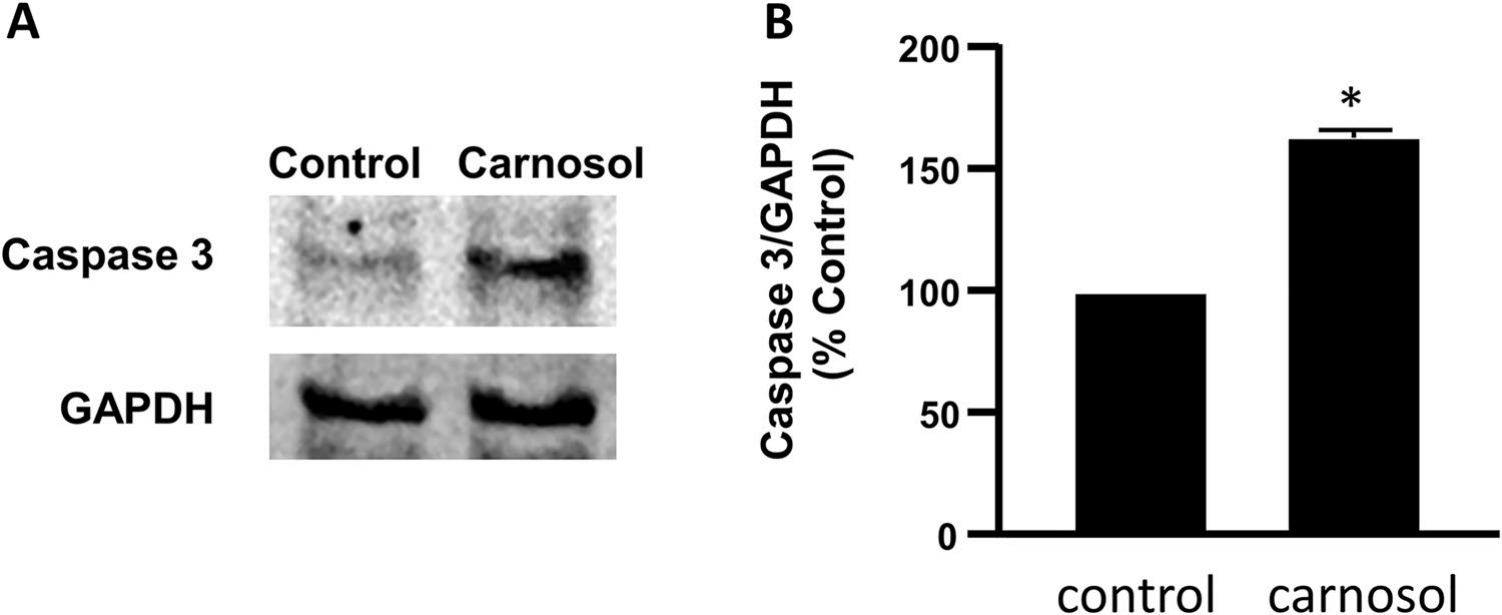
Effect of carnosol on caspase 3. (**A**) A typical image of Western blot analysis and (**B**) summarized data showed that carnosol (10^–4^ M, 24 h) significantly increased caspase-3 protein, indicating that carnosol increases cell apoptosis in FLO-1 cells. N = 4, * t-test, *P* < 0.05. Original blots are presented in Supplementary Figure 1.

We first examined whether carnosol decreases cell proliferation by using WST-1 cell proliferation assay. We found that carnosol dose-dependently decreased cell proliferation in FLO-1 esophageal adenocarcinoma cells (Fig. 1, ANOVA, *P* < 0.001). To further confirm this result, we performed Western blot analyses by using caspase-3 antibody. We found that carnosol significantly increased caspase-3 protein (Fig. 2, t test, *P* < 0.05), indicating that carnosol increases cell apoptosis in esophageal adenocarcinoma cells. These data suggest that carnosol decreases cell proliferation and increases cell apoptosis in FLO-1 cells.

### Reactive oxygen species (ROS) may mediate carnosol’s effect

**Figure 3:**
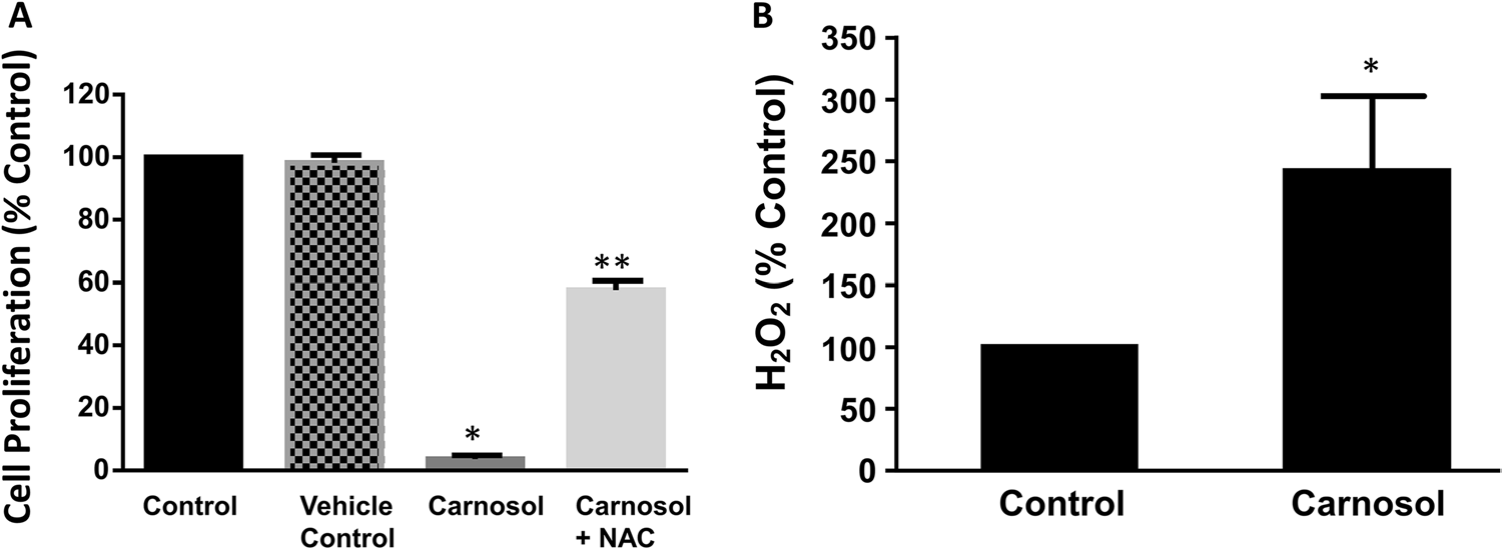
Reactive oxygen species (ROS) may mediate carnosol’s effect. (**A**) Pre-treatment with N-acetyl cysteine (NAC, 10^–6^ M), a ROS scavenger, for 1 h significantly inhibited carnosol-induced decrease in cell proliferation, indicating that ROS may mediate carnosol (10^–4^ M, 24 h)-induced decrease in cell proliferation. N = 3, ANOVA, *P < 0.001, compared with vehicle control; **P < 0.001 compared with carnosol. (**B**) Hydrogen peroxide in the culture medium was measured. Carnosol (10^–4^ M, 3 h) significantly increased H_2_O_2_ production, suggesting that ROS may contribute at least in part to carnosol’s effect. N = 3, *t test, *P* < 0.05.

Intracellular reactive oxygen species may play an important role in cell apoptosis. Therefore, we examined the effect of ROS on carnosol-induced decrease in cell proliferation by using N-acetyl cysteine (NAC), a ROS scavenger, on cell proliferation. As shown in Fig. 3A, pre-treatment with NAC for 1 h significantly inhibited carnosol-induced decrease in cell proliferation (ANOVA, *P* < 0.001), indicating that ROS may mediate carnosol-induced decrease in cell proliferation.

To confirm this result, we measured hydrogen peroxide in the culture medium. Cells were treated with vehicle or carnosol (10^–4^ M) for 3 h and then culture medium was collected for measurement. As shown in Fig. 3B, carnosol significantly increased H_2_O_2_ production (t test, *P* < 0.05), suggesting that ROS may contribute at least in part to carnosol’s effect.

### NADPH oxidases may be involved in carnosol’s effect

**Figure 4:**
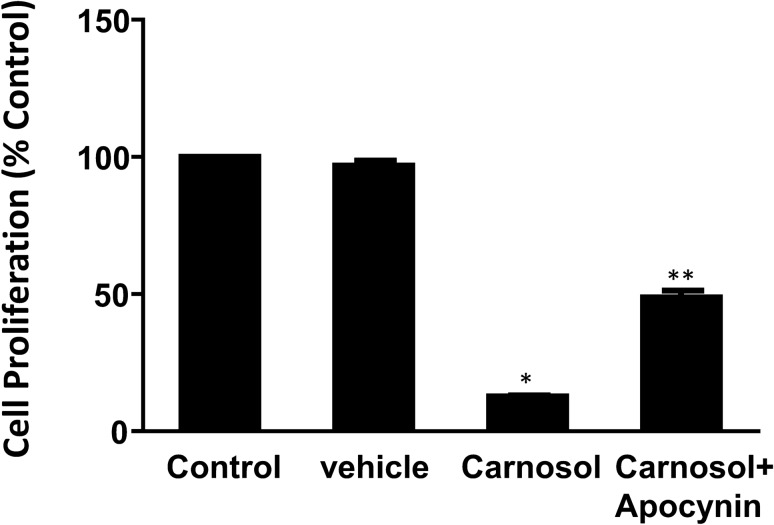
NADPH oxidases may be involved in carnosol’s effect. Carnosol (10^–4^ M, 24 h)-induced decrease in cell proliferation was partially reversed by NADPH oxidase inhibitor apocynin (10^–6^ M), suggesting that NADPH oxidases may be partially involved in carnosol’s effect. N = 12, ANOVA, *P < 0.0001, compared with vehicle control; **P < 0.0001 compared with carnosol.

We have shown that NADPH oxidases were present in FLO-1 cells^17^. Therefore, we examined the role of NADPH oxidases in carnosol-induced decrease in cell proliferation. Figure 4 showed that carnosol-induced decrease in cell proliferation was partially reversed by NADPH oxidase inhibitor apocynin (ANOVA, *P* < 0.0001), suggesting that NADPH oxidases may be partially involved in carnosol’s effect.

### SODD is involved in carnosol-induced decrease in cell proliferation in FLO-1 Cells.

**Figure 5:**
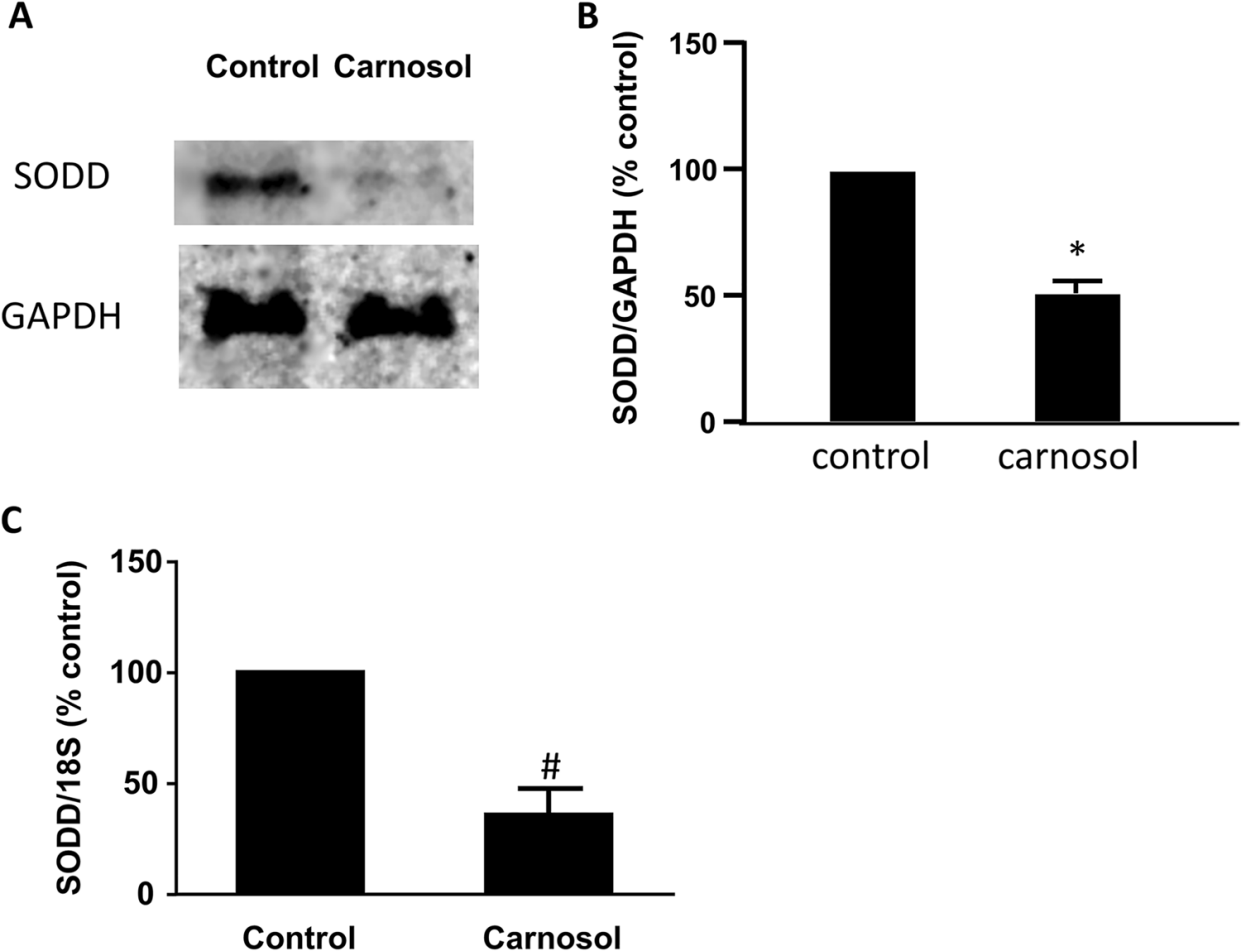
SODD is involved in carnosol-induced decrease in cell proliferation in FLO-1 cells. (**A**) A typical image of Western blot analysis and (**B**) summarized data showed that carnosol (10^–4^ M, 24 h) significantly decreased SODD protein, indicating that carnosol downregulates SODD protein in FLO-1 cells. N = 4, * t-test, *P* < 0.05. Original blots are presented in Supplementary Figure 2. (**C**) 24-h treatment with carnosol (10^–4^ M) significantly decreased SODD mRNA expression in FLO-1 cells. ^#^*P* = 0.001, t test, N = 8.

We have previously showed that acid-induced increase in SODD expression depends on the activation of NOX5-S and NF-κB1 p50 in FLO EA cells^18^. Therefore, we examined whether carnosol-induced decrease in cell proliferation is through the reduction of SODD. Figure 5 showed that 24-h treatment with carnosol (10^–4^ M) significantly decreased SODD protein (Fig. 5A,B, t test, *P* < 0.05) and mRNA expression (Fig. 5C, t test, *P* = 0.001), supporting our hypothesis that SODD may be involved in carnosol induced reduction in cell proliferation.

**Figure 6:**
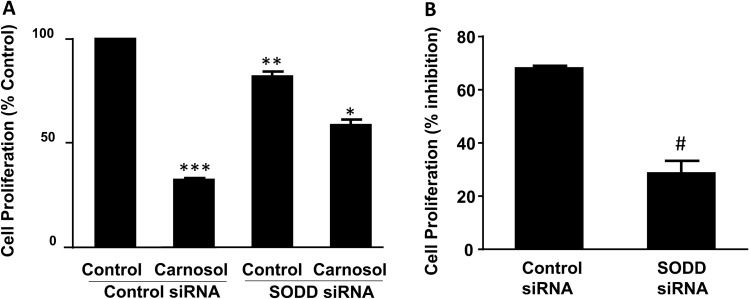
Role of SODD in carnosol-induced decrease in cell proliferation. (**A**) Knockdown of SODD at basal condition significantly decreased cell proliferation, indicating that SODD contributes to the cell proliferation at the basal condition in FLO-1 cells. Carnosol significantly decreased cell proliferation in cells transfected with control siRNA, a decrease which was partially reversed by knockdown of SODD. N = 3, ANOVA, ***P < 0.001, compared with control transfected with control siRNA, **P < 0.02, compared with control transfected with control siRNA; *P < 0.05, compared with carnosol transfected with control siRNA. B. In cells transfected with control siRNA, carnosol caused 68 ± 1% inhibition of cell proliferation. This inhibition was significantly reduced by knockdown of SODD (28.5 ± 4.8%). The data suggest that downregulation of SODD may mediate carnosol-induced reduction in cell proliferation. N = 3, t test, ^#^*P* < 0.02.

To further confirm this result, we used SODD siRNA to knock down SODD. We have shown that SODD siRNA successfully downregulated SODD protein expression^18^. Figure 6A showed that knockdown of SODD at basal condition significantly decreased cell proliferation (*P* < 0.02), indicating that SODD contributes to the cell proliferation at the basal condition in FLO-1 cells. In cells transfected with control siRNA, carnosol caused 68 ± 1% inhibition of cell proliferation (Fig. 6B). This inhibition was significantly reduced by knockdown of SODD (28.5 ± 4.8%, *P* < 0.02). The data suggest that downregulation of SODD may mediate carnosol-induced reduction in cell proliferation.

## Discussion

We found that carnosol inhibited cell proliferation and increased cell apoptosis in esophageal adenocarcinoma cells since carnosol dose-dependently decreased cell proliferation and significantly increased caspase-3 protein, suggesting that carnosol has anticancer activity in esophageal adenocarcinoma. The carnosol’s effect may be mediated by reactive oxygen species as supported by our results that N-acetyl cysteine (NAC) blocked carnosol-induced decrease in cell proliferation and carnosol significantly increased H_2_O_2_ production. This result is consistent with the results in breast cancer cells^16^ and human colon cancer HCT116^11^. In breast cancer cells^16^, carnosol activates ROS-dependent ER-stress responses, whereas in human colon cancer cells carnosol-induced apoptosis is mediated by inactivation of STAT3 through the production of ROS. Reactive oxygen species have dual effects. Low doses of ROS activate cell survival signaling pathway, whereas high doses of ROS induce cell apoptosis^19^. Therefore, carnosol might induce ROS production high enough to cause cell apoptosis.

ROS may be generated in mitochondria through the respiratory chains or by enzymes such as NADPH oxidases. Apocynin is a naturally occurring methoxy-substituted catechol and is an inhibitor of NADPH-oxidase^20–22^. It reduces the production of superoxide from activated neutrophils and macrophages, but the exact mechanism of its inhibition is not fully understood^23^. It is thought that apocynin inhibits reactive oxygen species (ROS) by inhibition of the assembly of NADPH-oxidase^23^. We found that apocynin partially reversed carnosol-induced decrease in cell proliferation, suggesting that NADPH oxidases might be partially involved in carnosol’s effect.

SODD is an anti-apoptotic protein and belongs to the BAG family^24^. SODD has been reported to be increased in cancer tissues; e.g. pancreatic cancers^25^, an increase of SODD expression which may decrease cell death and increase cell proliferation in various cancer cell lines^25,26^. SODD is associated with the ATPase domain of Hsc70/Hsp70^24^, the cytoplasmic domain of the tumor necrosis factor receptor 1 (TNFR1) and death receptor-3. TNFR1 signaling complex is activated by the release of SODD from TNFR1, which permits the recruitment of TNFR-associated death domain and TNFR-associated factor 2^26^. SODD inhibits cell apoptosis through its binding to TNFR1 and preventing the ligand-independent oligomerization and spontaneous activation of TNFR1^27^. We found that carnosol significantly decreased SODD protein and mRNA expression, indicating that carnosol may decrease cell proliferation via the downregulation of SODD. This result was further confirmed by the knockdown study showing that knockdown of SODD significantly blocked carnosol-induced inhibition of cell proliferation.

Carnosol might also play its anticancer role via epigenetic changes. For example, carnosol has been shown to inhibit p300 histone acetyl transferase activity by blocking the acetyl-CoA binding to the histone acetyl transferase catalytic domain since carnosol occupies the region where the pantetheine arm of the acetyl-CoA is bound^15^. It is well known that p300 is a coactivator of multiple transcription factors related to many biological processes^28^. High levels of p300 are associated with breast cancer progression^29^ and poor prognosis^30^. The inhibition of p300 histone acetyl transferase will decrease p300 acetylation, thus increasing p300 degradation. Whether this epigenetic modification of p300 is involved in carnosol’s anticancer effect in esophageal adenocarcinoma needs to be further explored.

In conclusion, carnosol dose-dependently decreased cell proliferation and significantly increased caspase-3 protein. Carnosol’s effect may be through the overproduction of ROS and the downregulation of SODD. Carnosol may be useful for the treatment of esophageal adenocarcinoma.

## Materials and methods

### Cell culture and treatment

The human Barrett’s adenocarcinoma cell line FLO-1^31^ was obtained from Dr. David Beer (University of Michigan). The FLO-1 cells were cultured in Dulbecco’s modified Eagle’s medium (DMEM) containing 10% fetal bovine serum and antibiotics.

### Cell proliferation assay

Cell proliferation was determined by using WST-1 Cell Proliferation Assay. Cell Proliferation Reagent WST-1 was purchased from MilliporeSigma. 2 × 10^4^ FLO-1 cells were seeded in a 96-well plate. When cells reached 50–60% confluence, different concentrations of carnosol or vehicle controls were added to each well. After cells were cultured for 24 h, 10 μl WST-1 reagent was added to each well and cells were cultured for additional 1 h. Then the absorbance of each well was determined by using a BioTek Synergy H4 hybrid reader at 440 nm. Medium without cells was used as a blank. Carnosol was dissolved in ethanol and the same concentrations of ethanol were used in the control groups. The experiments were repeated three times for the experiments of siRNA, N-acetyl cysteine and carnosol’s effects on cell proliferation and twelve times for apocynin experiments.

### Amplex® red hydrogen peroxide fluorescent assay

Levels of H_2_O_2_ in culture medium were measured by using Amplex® Red H_2_O_2_ Assay Kit (Thermo Fisher Scientific Inc., Waltham, MA) as we previously described^32^. The assay was performed in triplicates.

### Small interfering RNA(SiRNA) transfection

FLO-1 cells were transfected with control siRNA or SODD siRNA by using Lipofectamine 2000 (Invitrogen, Grand Island, New York, USA) according to the manufacturer’s instruction and as we previously described^18^. 24 h after transfection, FLO-1 cells were treated with vehicle or carnosol (10^–4^ M) for additional 24 h.

### Reverse-transcription PCR and quantitative real-time PCR

Reverse-transcription PCR and quantitative real-time PCR were performed as we previously described^18^. The primers used were as follows: SODD forward, 5′-GGGGTACCCAATGGTGCGATCTCGGCTCACTG-3′; SODD reverse, 5′-GAAGATCTCTCGAGGGGATCCGCTGCCCTGAAGCGCT-3′; 18S forward, 5′-CGGACAGGATTGACAGATTGATAGC-3′; and 18S reverse, 5′-TGCCAGAGTCTCGTTCGTTATCG -3′. The experiments were repeated eight times.

### Western blot analysis

FLO-1 cells were treated with carnosol (10^−4^ M) or vehicle for 24 h and then collected for Western blot analysis. Western blot analysis was performed as described previously^33^. Primary antibodies used were as follows: SODD antibody (1:1000, Santa Cruz biotechnologies), caspase-3 antibody (1:2000, Upstate Biotechnology, Waltham, MA) and GAPDH antibody (1:2000, Santa Cruz biotechnologies). The experiments were performed in quadruplets.

### Materials

Carnosol and N-acetyl cysteine (NAC) were purchased from Cayman Chemical Co. (Ann Arbor, MI, USA). Apocynin was purchased from Millipore Sigma (Burlington, MA).

### Statistical analysis

All statistical analyses were performed by using GraphPad Prism software version 6 (GraphPad Software, Inc., Boston, MA). Data were expressed as mean ± S.E. Statistical differences between two groups were determined by Student’s *t* test. Differences among multiple groups were tested using analysis of variance (ANOVA) and checked for significance using Fisher’s protected least significant difference test.

## Supplementary Information

Supplementary Information.

## Data Availability

All data generated or analyzed during this study are included in this published article and its supplementary information file.

## References

[1] Blot WJ, Devesa SS, Kneller RW, Fraumeni JF (1991). Rising incidence of adenocarcinoma of the esophagus and gastric cardia. JAMA.

[2] Pennathur A, Gibson MK, Jobe BA, Luketich JD (2013). Oesophageal carcinoma. Lancet.

[3] Thrift AP, Whiteman DC (2012). The incidence of esophageal adenocarcinoma continues to rise: analysis of period and birth cohort effects on recent trends. Ann. Oncol..

[4] Lagergren J, Bergstrom R, Lindgren A, Nyren O (1999). Symptomatic gastroesophageal reflux as a risk factor for esophageal adenocarcinoma. N. Engl. J. Med..

[5] Wild CP, Hardie LJ (2003). Reflux, Barrett's oesophagus and adenocarcinoma: Burning questions. Nat. Rev. Cancer.

[6] Souza RF, Morales CP, Spechler SJ (2001). Review article: a conceptual approach to understanding the molecular mechanisms of cancer development in Barrett's oesophagus. Aliment. Pharmacol. Ther..

[7] Falk GW (2002). Barrett's esophagus. Gastroenterology.

[8] Crane SJ (2008). Survival trends in patients with gastric and esophageal adenocarcinomas: a population-based study. Mayo Clin. Proc..

[9] Sihvo EI, Luostarinen ME, Salo JA (2004). Fate of patients with adenocarcinoma of the esophagus and the esophagogastric junction: a population-based analysis. Am. J. Gastroenterol..

[10] Hur C (2013). Trends in esophageal adenocarcinoma incidence and mortality. Cancer.

[11] Park KW (2014). Carnosol induces apoptosis through generation of ROS and inactivation of STAT3 signaling in human colon cancer HCT116 cells. Int. J. Oncol..

[12] Alsamri H (2019). Carnosol, a natural polyphenol, inhibits migration, metastasis, and tumor growth of breast cancer via a ROS-dependent proteasome degradation of STAT3. Front. Oncol..

[13] Wang L (2018). Carnosol suppresses patient-derived gastric tumor growth by targeting RSK2. Oncotarget.

[14] Johnson JJ (2011). Carnosol: A promising anti-cancer and anti-inflammatory agent. Cancer Lett..

[15] Alsamri H (2021). Carnosol is a novel inhibitor of p300 acetyltransferase in breast cancer. Front. Oncol..

[16] Alsamri H (2022). Carnosol induces p38-mediated ER stress response and autophagy in human breast cancer cells. Front. Oncol..

[17] Hong J (2011). Role of Rac1 in regulation of NOX5-S function in Barrett's esophageal adenocarcinoma cells. Am. J. Physiol. Cell Physiol..

[18] Li D, Hong J, Cao W (2017). Silencer-of-death domain mediates acid-induced decrease in cell apoptosis in Barrett's associated esophageal adenocarcinoma cells. J. Pharmacol. Exp. Ther..

[19] Redza-Dutordoir M, Averill-Bates DA (1863). Activation of apoptosis signalling pathways by reactive oxygen species. Biochim. Biophys. Acta.

[20] Lafeber FP (1999). Apocynin, a plant-derived, cartilage-saving drug, might be useful in the treatment of rheumatoid arthritis. Rheumatology (Oxford).

[21] Zhang Y (2005). Apocynin but not allopurinol prevents and reverses adrenocorticotropic hormone-induced hypertension in the rat. Am. J. Hypertens..

[22] Stolk J, Hiltermann TJ, Dijkman JH, Verhoeven AJ (1994). Characteristics of the inhibition of NADPH oxidase activation in neutrophils by apocynin, a methoxy-substituted catechol. Am. J. Respir. Cell Mol. Biol..

[23] Stefanska J, Pawliczak R (2008). Apocynin: Molecular aptitudes. Mediators Inflamm.

[24] Takayama S, Xie Z, Reed JC (1999). An evolutionarily conserved family of Hsp70/Hsc70 molecular chaperone regulators. J. Biol. Chem..

[25] Ozawa F, Friess H, Zimmermann A, Kleeff J, Buchler MW (2000). Enhanced expression of Silencer of death domains (SODD/BAG-4) in pancreatic cancer. Biochem. Biophys. Res. Commun..

[26] Jiang Y, Woronicz JD, Liu W, Goeddel DV (1999). Prevention of constitutive TNF receptor 1 signaling by silencer of death domains. Science.

[27] Doong H, Vrailas A, Kohn EC (2002). What's in the 'BAG'?—A functional domain analysis of the BAG-family proteins. Cancer Lett..

[28] Chen J, Li Q (2011). Life and death of transcriptional co-activator p300. Epigenetics.

[29] Fermento ME (2014). Inhibition of p300 suppresses growth of breast cancer. Role of p300 subcellular localization. Exp. Mol. Pathol..

[30] Xiao XS (2011). High expression of p300 in human breast cancer correlates with tumor recurrence and predicts adverse prognosis. Chin. J. Cancer Res..

[31] Hughes SJ (1997). Fas/APO-1 (CD95) is not translocated to the cell membrane in esophageal adenocarcinoma. Cancer Res..

[32] Si J (2007). NADPH oxidase NOX5-S mediates acid-induced cyclooxygenase-2 expression via activation of NF-kappaB in Barrett's esophageal adenocarcinoma cells. J. Biol. Chem..

[33] Cao W (2003). MAPK mediates PKC-dependent contraction of cat esophageal and lower esophageal sphincter circular smooth muscle. Am. J. Physiol. Gastrointest. Liver Physiol..

