# Supplementary material for: Downregulation of SODD mediates carnosol-induced reduction in cell proliferation in esophageal adenocarcinoma cells
Source: Sci Rep. 2023 Jun 29;13:10580. doi: 10.1038/s41598-023-37796-5 (PMC10310760; doi:10.1038/s41598-023-37796-5)

Downregulation of SODD mediates carnosol-induced reduction in cell proliferation in esophageal adenocarcinoma cells

Aihua Li[1,2], Weibiao Cao[1]

[1] Department of Pathology and Laboratory Medicine, Rhode Island Hospital and The Alpert Medical School of Brown University, Providence, RI; [2] Department of Gastroenterology, Chongqing University Cancer Hospital, Chongqing, China

Supplementary figure 1 (Figure 2 in the manuscript)

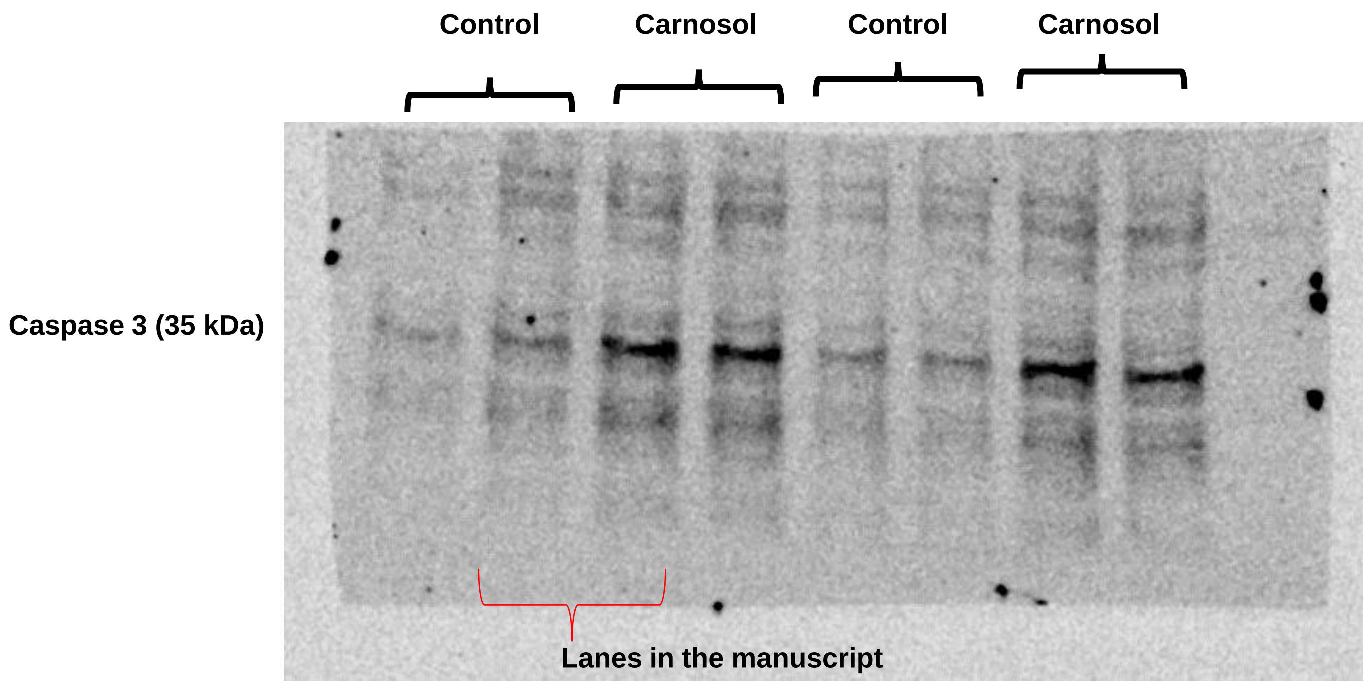

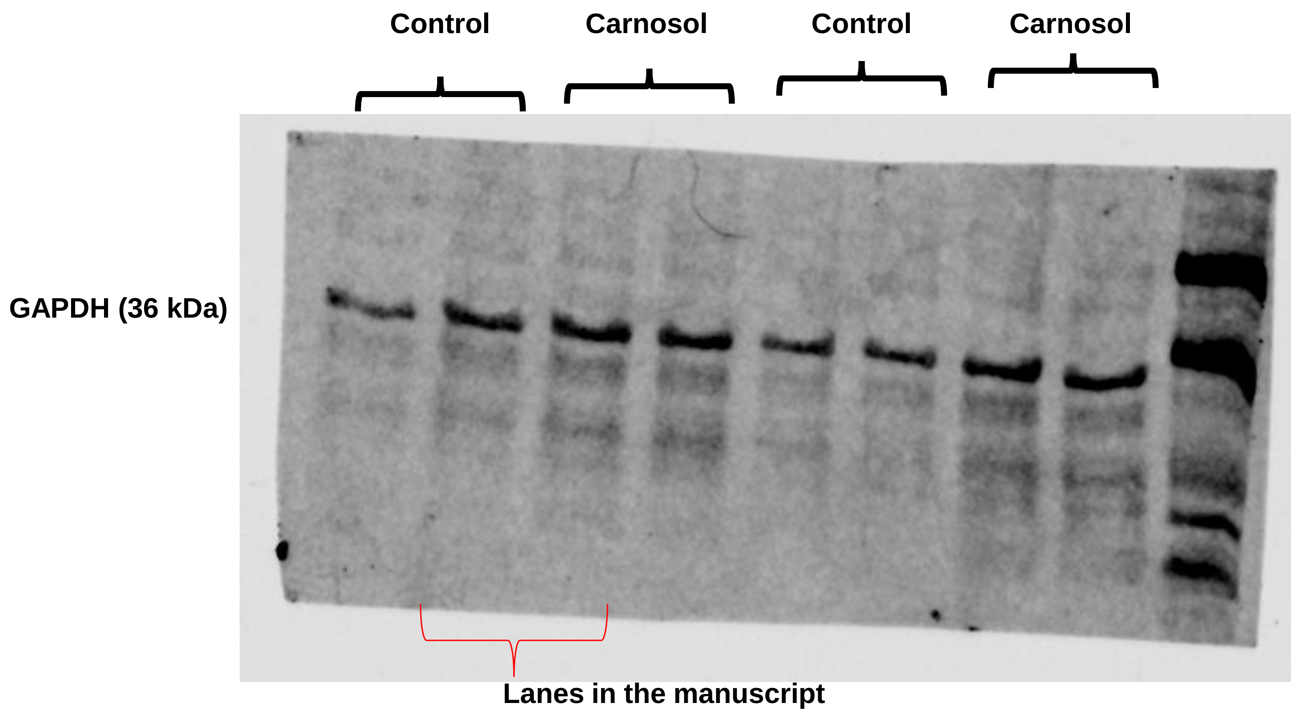

Supplementary figure 2 (Figure 5 in the manuscript)

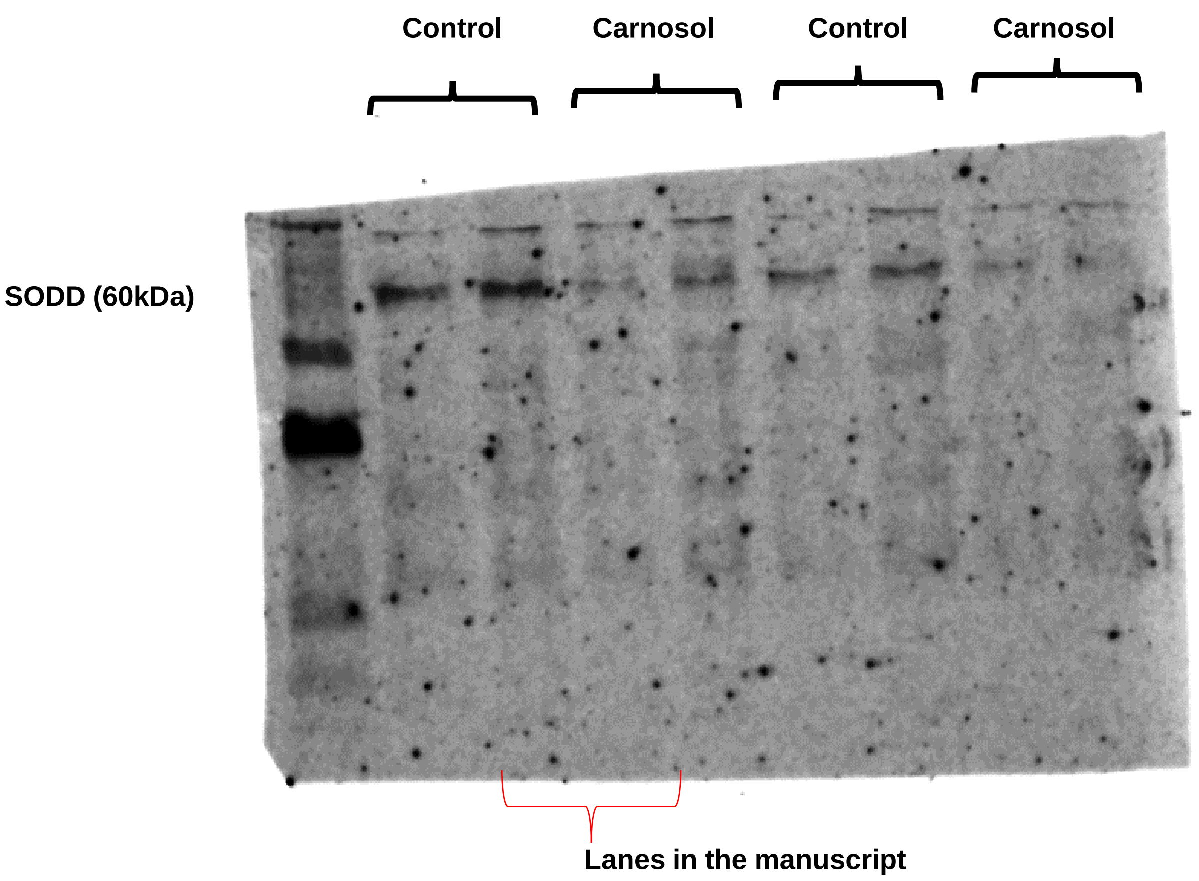

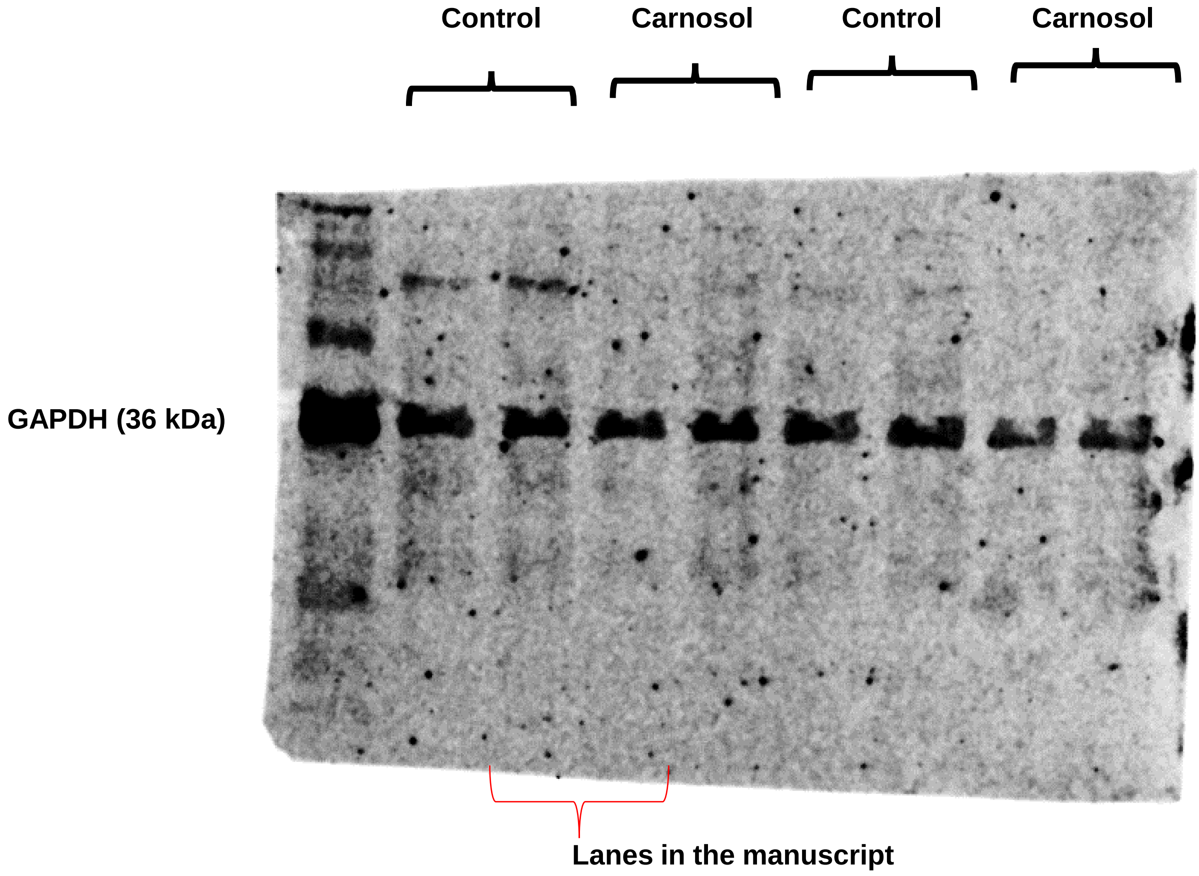

Supplement: Supplementary file 1 — Supplementary Information. [file 41598_2023_37796_MOESM1_ESM.pdf]
